# Combined diffusion-weighted and functional magnetic resonance imaging reveals a temporal-occipital network involved in auditory-visual object processing

**DOI:** 10.3389/fnint.2013.00005

**Published:** 2013-02-13

**Authors:** Anton L. Beer, Tina Plank, Georg Meyer, Mark W. Greenlee

**Affiliations:** ^1^Institut für Psychologie, Universität RegensburgRegensburg, Germany; ^2^Experimental and Clinical Neurosciences Programme, Universität RegensburgRegensburg, Germany; ^3^Department of Experimental Psychology, University of LiverpoolLiverpool, UK

**Keywords:** multisensory processing, auditory cortex, superior temporal sulcus, extrastriate body area, fMRI, DWI, structural connectivity, fiber tractography

## Abstract

Functional magnetic resonance imaging (MRI) showed that the superior temporal and occipital cortex are involved in multisensory integration. Probabilistic fiber tracking based on diffusion-weighted MRI suggests that multisensory processing is supported by white matter connections between auditory cortex and the temporal and occipital lobe. Here, we present a combined functional MRI and probabilistic fiber tracking study that reveals multisensory processing mechanisms that remained undetected by either technique alone. Ten healthy participants passively observed visually presented lip or body movements, heard speech or body action sounds, or were exposed to a combination of both. Bimodal stimulation engaged a temporal-occipital brain network including the multisensory superior temporal sulcus (msSTS), the lateral superior temporal gyrus (lSTG), and the extrastriate body area (EBA). A region-of-interest (ROI) analysis showed multisensory interactions (e.g., subadditive responses to bimodal compared to unimodal stimuli) in the msSTS, the lSTG, and the EBA region. Moreover, sounds elicited responses in the medial occipital cortex. Probabilistic tracking revealed white matter tracts between the auditory cortex and the medial occipital cortex, the inferior occipital cortex (IOC), and the superior temporal sulcus (STS). However, STS terminations of auditory cortex tracts showed limited overlap with the msSTS region. Instead, msSTS was connected to primary sensory regions via intermediate nodes in the temporal and occipital cortex. Similarly, the lSTG and EBA regions showed limited direct white matter connections but instead were connected via intermediate nodes. Our results suggest that multisensory processing in the STS is mediated by separate brain areas that form a distinct network in the lateral temporal and inferior occipital cortex.

## Introduction

Identifying objects or actions in our environment usually relies on multiple sources of sensory cues such as sounds and images. Over the last decade several functional magnetic resonance imaging (fMRI) studies have associated the superior temporal cortex (STC) with multisensory object and action processing. For instance, reliable blood-oxygenation-level-dependent (BOLD) responses to auditory and visual stimuli as well as enhanced BOLD responses to bimodal stimuli were observed in a posterior part of the superior temporal sulcus (STS) (Beauchamp et al., 2004b, 2008; Hein et al., 2007; Noesselt et al., 2007). The authors suggested that the multisensory STS (msSTS) region found in humans likely reflects a homologue of the polysensory STS region observed in macaques. As brain imaging techniques such as fMRI, electroencephalography (EEG), or magnetoencephalography (MEG) detect the activity of large neural ensembles, overlapping (or enhanced) responses may also result from separate but interspersed neural populations rather than reflecting multisensory integration. Therefore, several researchers examined violations from linear summation or race model violations in brain or behavioral responses to bimodal stimuli as such violations suggest multisensory interactions (Schröger and Widmann, 1998; Laurienti et al., 2005; Stein et al., 2009; but see also Gondan and Röder, 2006; Proctor and Meyer, 2011; Szameitat et al., 2011). For instance, (degraded) bimodal auditory-visual stimuli elicited larger BOLD responses in msSTS than predicted by the sum of the BOLD responses to corresponding unimodal stimuli (Calvert et al., 2000; Werner and Noppeney, 2010b). However, such “superadditive” responses are not always observed (Hocking and Price, 2008; Meyer et al., 2011) and likely require degraded or noise stimuli (Laurienti et al., 2005; Angelaki et al., 2009). Other studies adopting salient (non-degraded) bimodal stimuli observed “subadditive” EEG/MEG responses (Schröger and Widmann, 1998) with a source in the STS (Raij et al., 2000; Cappe et al., 2010).

Other researchers compared responses to synchronous (simultaneously presented) and asynchronous (presented with a temporal offset) auditory-visual stimulus pairs (Calvert et al., 2000; Miller and D'Esposito, 2005; Noesselt et al., 2007, 2012; Stevenson et al., 2011). They found that synchronous stimulus pairs (that were perceived as fused) elicited stronger BOLD signals in the STS than asynchronous pairs. These findings suggest that the STS merges multimodal signals based on temporal synchrony. Other studies examined semantic congruency between pairs of object sounds and visual stimuli (Hocking and Price, 2008). For instance, semantically incongruent auditory-visual stimulus pairs elicited more pronounced MEG responses in the STS compared to that evoked by congruent pairs (Raij et al., 2000). Similarly, fMRI adaptation research found that incongruent pairs of syllable sounds and lip movies that elicited the well-known McGurk illusion (McGurk and MacDonald, 1976) were associated with more adaptation in the STS than auditory-visual pairs that failed to elicit the McGurk illusion (Benoit et al., 2010). Likewise, video clips of point-light lip or body movements elicited weaker BOLD signals in the posterior STS when paired with congruent speech sounds or body action sounds, respectively, than when paired with incongruent sounds (Meyer et al., 2011). In this latter study, a one-back task was adopted which required observers to memorize a representation of the multimodal stimuli. Moreover, the BOLD difference between congruent and incongruent sound-video pairs was only observed with stimuli of real objects and actions but not with noise stimuli. This suggests that the STS contributes to a supramodal representation of objects and actions based on converging input of auditory and visual signals.

Many studies imply that multisensory processing relies on a single region within the posterior STS. However, recent progress in fMRI research (Beauchamp et al., 2004a; Van Atteveldt et al., 2010) and cell recordings (Dahl et al., 2009) suggests that multisensory processing in the STC relies on a network of spatially distinct regions and that the STC shows a more patchy organization than previously thought. For instance, processing of multimodal synchrony seems to involve at least two distinct sub-parts of the STS (Stevenson et al., 2011; Noesselt et al., 2012). Congruent compared to incongruent auditory-visual motion stimuli elicited more pronounced BOLD responses in the superior temporal gyrus (STG)—rather than the STS (Baumann and Greenlee, 2007). Likewise, spatially-semantic congruent sound-picture pairs elicited more activity in the STG compared to that evoked by incongruent sound-picture pairs (Plank et al., 2012). These lateral STG regions likely correspond to lateral belt and parabelt regions of the auditory cortex as described in macaques (Petkov et al., 2006) rather than the msSTS. Regions relevant for multisensory object processing were also observed outside the STS/STG complex. Several studies showing multisensory responses in the STS also reported multisensory activity in the inferior occipito-temporal cortex, anterior insula, and ventrolateral frontal cortex (Calvert et al., 2000; Beauchamp et al., 2004b; Hein et al., 2007; Meyer et al., 2011; Nath and Beauchamp, 2012). This diversity of findings suggests that the notion of a unitary STS region related to multisensory object processing needs to be re-considered. It is likely that multisensory object processing relies on separate but inter-connected brain areas within the STC, the inferior occipito-temporal cortex and the frontal lobe. If this network notion is true, then understanding the connections between the nodes of this network becomes crucial in understanding multisensory processing.

Diffusion weighted imaging (DWI), first described in 1985 (Le Bihan and Breton, 1985) and sometimes also referred to as diffusion tensor imaging (Basser et al., 1994), is a non-invasive MRI technique that is sensitive to the diffusion of molecules (primarily water) in the brain. Molecular diffusion is primarily caused by thermal activity and is restricted by cell membranes. Brain regions containing coherent cell structures (e.g., axons of white matter) show a higher degree of anisotropic diffusion than other brain parts (e.g., somas and dendrites in gray matter). Voxel-wise measures of diffusion parameters such as the fractional anisotropy (FA) or diffusion vectors (tensors) derived from DWI allow inferences about the white matter structure. About one decade ago, tractographic approaches emerged that infer the path of least diffusion hindrance (tracks) across the white matter based on the diffusion parameters of an assembly of voxels. These white matter tracks are likely formed by axonal fiber bundles (tracts). Therefore, DWI-based tractography allows inferences about the white matter architecture of healthy humans or patients *in vivo* (Conturo et al., 1999; Jones et al., 1999; Mori et al., 1999; Lee et al., 2005). For instance, we recently reported evidence for white matter tracts between human auditory and visual cortex (Beer et al., 2011b). Combining tracking approaches based on DWI with conventional fMRI may resolve ambiguities in brain connectivity research. For instance, concurrent functional activity or resting-state connectivity between multiple brain areas do not necessarily require a direct (monosynaptic) anatomical connection (Damoiseaux and Greicius, 2009). Instead functional connectivity may result from indirect (polysynaptic) white matter connections. Moreover, structural connectivity studies have shown that brain areas, which cannot be distinguished otherwise, may be classified by their “connectivity fingerprints” (Behrens and Sporns, 2012).

The goal of this study was to examine the structural connections of the brain network involved in auditory-visual processing by means of white matter tracking. Therefore, probabilistic fiber tracking based on DWI was performed between auditory cortex and several brain areas involved in auditory-visual processing. We were primarily interested in the connectivity profile of the msSTS and related brain areas involved in processing biological sounds and visual motion. Brain areas involved in multisensory processing of speech and body actions were localized by whole-brain fMRI. The stimuli were adapted from a previous study that showed robust activation patterns in multisensory processing areas (Meyer et al., 2011). In order to control for confounds by behavioral responses, stimuli were task-irrelevant for the observer. Observers' attention was controlled by a simple detection task. Multisensory interactions were examined by a region-of-interest (ROI) analysis.

## Materials and methods

### Participants

The study comprised ten healthy volunteers (including one author, 7 females, all but one right-handed). All participants reported normal or corrected-to-normal vision and no hearing impairments. Their mean age was 27 years (range from 23 to 40). All participants gave written informed consent prior to the study. The procedure was approved by the ethical board of the University of Regensburg.

### Auditory-visual task

**Figure 1:**
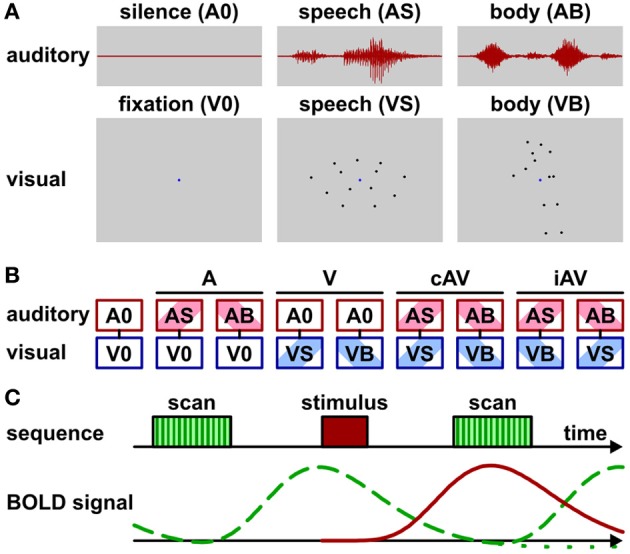
**Stimuli, conditions, and scan protocol. (A)** Schematic depiction of stimulus types. Auditory stimuli (1200 ms duration) were speech sounds (AS) such as “aba” or sounds generated by body movements (AB) such as “sawing” noise. No sound was presented during baseline conditions (A0). Visual stimuli consisted of video clips (1200 ms duration) with point-light (13 dots) lip (speech) (VS) or body movements (VB). A blue dot in the display center served as fixation marker. Only the fixation dot was shown during baseline condition (V0). See also Supplementary Movies 1 and 2. **(B)** Audio-visual stimulus combinations: baseline (fixation without sound, A0V0), auditory only conditions (ASV0 or ABV0), visual only conditions (A0VS or A0VB), semantically congruent (cAV) conditions (ASVS or ABVB), semantically incongruent (iAV) conditions (ASVB or ABVS). **(C)** Trial sequence and schematic BOLD signals for sparse imaging. MR scan (acquisition) time (TA) was 2070 ms with a repetition time (TR) of 8000 ms. Stimuli were presented 4500 ms after scan onset during acquisition-free periods. Note that the stimulus-induced BOLD signal (red solid line) reaches its maximum during the acquisition time. The BOLD signal induced by the scanner noise (green dashed line) drops to about baseline at the acquisition time. Its post-stimulus undershoot (green dotted line) is masked by the peak of the subsequent BOLD response.

Unisensory visual and auditory as well as multisensory (auditory-visual) brain areas were identified by fMRI while participants passively perceived biological motion stimuli (Figure 1A; Supplementary Movies 1 and 2). Task-irrelevant stimulus presentation was chosen in order to reduce the possibility that brain activity related to multisensory processing (e.g., in frontal cortex) was confounded by activity elicited by behavioral responses (see also Hein et al., 2007). Attention was controlled by asking observers to perform a simple detection task (see below). The stimuli were adopted from previous work in which they elicited significant activation in multisensory brain areas of the STC and other parts of the cortex (Meyer et al., 2011). In particular, visual stimuli consisted of videos with point-light displays of speech (lip) (VS) or body (VB) movements. Auditory stimuli consisted of sounds corresponding to the speech (AS) (sounds generated by the lip movements) or the body (AB) movements (sounds generated by the action). Speech stimuli represented nine distinct vowel-consonant-vowel syllables such as “aba” or “igi” spoken by a male speaker. Body stimuli represented nine distinct actions such as a person walking, jumping, cycling, rowing, sawing, etc. Each stimulus was presented for 1.2 s.

Each video clip showed 13 black moving dots on a gray background with a frame rate of 30 Hz. In addition, a blue dot in the center of the display served as a fixation point. The display size was normalized so that the mean dot deviation from the screen center was 2.7 degrees of visual angle (with a dot size of approximately 0.15 degrees). Participants viewed the video clips via a back-mirror (mounted within the head-coil) on a translucent screen positioned about 70 cm distant to the eye. Participants had to view the video clips while fixating the fixation point and press a button with their index finger whenever they detected a red dot. Accordingly, in some videos one dot was colored red for 300 ms during an interval between 300 and 900 ms. Sounds were presented via MR compatible headphones (MR confon, Magdeburg, Germany). Sound onset and offset was synchronized with the onset and offset of the video clips. All sounds were matched in root mean square power and presented with a sound pressure level of approximately 65 dB. Participants were asked to listen to the sounds and press a button whenever they detected a beep sound. Accordingly, in some trials a target beep sound (500 Hz, 130 ms, about 70 dB) occurred between 300 and 900 ms while the body action or speech sound was presented.

Visual and auditory stimuli were presented in nine conditions (Figure 1B) that were grouped into five main conditions: purely visual presentation of speech or body movements (VS or VB), purely auditory presentation of speech or body sounds (AS or AB), congruent auditory-visual presentation of speech or body stimuli (ASVS or ABVB), incongruent auditory-visual speech or body stimuli (ASVB or ABVS), or a silent baseline condition requiring participants to view the fixation point on an otherwise blank screen (A0V0). During congruent trials, each video clip was presented together with its matching sound. During incongruent trials, each clip was combined with a sound of the other category (e.g., speech sound with body action video).

In order to monitor attention, participants were asked to respond to a visual (red dot) or auditory (beep) target occurring in 20% of the trials (10% for each target). Targets were balanced across all stimulus conditions. They occurred in a pseudo-random order and never occurred on the same trial. The participants pressed a button on a response box upon target detection.

Stimuli were presented with an inter-trial-interval (ITI) of 8000 ms. This relatively large ITI was required, because a sparse-imaging MRI acquisition protocol with a repetition time (TR) of 8000 ms and an acquisition time (TA) of 2070 ms was adopted (Figure 1C). Sparse-imaging successfully reduces the influence of the scanner noise on the BOLD response (see Edmister et al., 1999; Engelien et al., 2002). Stimuli started 4.5 s after the onset (2.43 s after the offset) of the scanner acquisition phase. This timing was chosen for two reasons: First, in order to assure adequate perception, stimuli were presented during the silent phase of the functional measurement stream. Second, based on previous experience the BOLD response reaches its maximum about 3–5 s after stimulus onset. A TR of 8 s and a stimulus onset at 4.5 s assured that the MR acquisition following the stimulus best captured the stimulus-induced BOLD signal with little interference by the BOLD signal induced by the scanner noise. Note that this timing ignores the “post-stimulus undershoot”—a temporary decline below baseline following the main peak—of the scanner noise BOLD response. As this post-stimulus undershoot is substantially smaller in magnitude than the main peak, equal across measurements, and masked by the main peak of subsequent measurements, it is usually ignored in auditory fMRI research (e.g., Petkov et al., 2006; Benoit et al., 2010). Each run of the multisensory task lasted about 12 min and consisted of 90 trials. At least three runs were conducted for each participant. Trials were pseudo-randomized in each run to avoid carry-over effects. Trial number per condition was balanced within each run. Stimulus pairings (e.g., targets combined with the different stimuli: “aba,” “igi,”…) were balanced across runs.

### Data acquisition

All MRI data was acquired by a 3T head-only Allegra scanner (Siemens, Erlangen, Germany) using a one-channel whole-head coil while participants laid supine (head first) in the scanner bore. Head motion during the scans was constrained by cushions. For each participant, one high-resolution structural run, a series of functional runs, and three diffusion-weighted runs were acquired. The structural images were acquired with a magnetization-prepared rapid acquisition gradient echo (MPRAGE) sequence (TR = 2250 ms, echo time = 2.6 ms, inversion time = 900 ms, flip angle = 9°). The parameters were adapted from the Alzheimer's disease Neuroimaging project (Laboratory for Neuro Imaging, UCLA, Los Angeles, CA). The 160 sagittal slices covered the whole brain (voxel size = 1 × 1 × 1 mm^3^, field of view = 256 × 256 mm^2^). Functional runs were acquired with a T2^*^ weighted echoplanar sparse-imaging sequence (TR = 8000 ms, TA = 2070 ms, echo time = 30 ms, flip angle = 90°) with 36 axial slices (voxel size = 3 × 3 × 3 mm^3^, field of view = 192 × 192 mm^2^, no inter-slice gap, interleaved acquisition). DWI runs were acquired with a single-shot pulsed gradient spin-echo sequence with echoplanar readout (TR = 7200 ms, echo time = 95 ms, flip angle = 90°). Diffusion was examined along 30 isotropically distributed orientations (Jones et al., 2002) and weighted by a b-value of 1000 s/mm^2^. Five volumes without diffusion weighting (b-value of zero) were interspersed into the diffusion sequence every six volumes. The 54 axial slices covered the whole brain (voxel size = 2.5 × 2.5 × 2.5 mm^3^, field of view = 240 × 240 mm^2^).

### Cortical reconstruction

Cortical reconstruction was automatically performed with Freesurfer version 4.1 (Martinos Center for Biomedical Imaging, Charlestown, MA) as described elsewhere (Beer et al., 2009). In brief, non-brain tissue was removed (Segonne et al., 2004), images were intensity corrected and normalized (Sled et al., 1998), subcortical volumetric structures were segmented (Fischl et al., 2002, 2004a), and the gray-white matter boundary was tessellated and topologic inaccuracies automatically corrected (Fischl et al., 2001; Segonne et al., 2007). Then, the cortical surface was deformed (Dale et al., 1999), inflated (Fischl et al., 1999a), registered to a spherical atlas that preserves individual folding patterns to match the cortical geometry across subjects (Fischl et al., 1999b), and automatically parcellated into units based on gyral and sulcal structures (Fischl et al., 2004b; Desikan et al., 2006).

### Whole-brain analysis of fMRI

The fMRI data was analyzed with the FSFAST tools of Freesurfer. Pre-processing included motion correction (to the first volume of each session), intensity normalization (Cox and Jesmanowicz, 1999), and spatial smoothing with a three-dimensional Gaussian kernel of 8 mm (full-width at half-maximum). The first volume of each session was automatically co-registered to the structural volume. All co-registrations were verified by blink comparison and manually corrected if necessary.

In order to define brain regions relevant for auditory-visual processing, we performed a general linear model (GLM) whole-brain group analysis. The design matrix of the GLM contained separate predictors for all nine conditions (see Figure 1B) and a second order polynomial to model MR signal drift artifacts. Note that because of the sparse-imaging protocol (TR = 8000 ms, TA = 2070 ms) only one acquisition following stimulus presentation was modeled by a box-car predictor. In order to maximize statistical power and to detect all relevant brain regions, target trials (10% beep or 10% red dot) were included in the whole-brain analysis. A control analysis excluding these trials (not reported here) showed similar results. Note that target trials were excluded from the functional ROI analysis (see below). Group statistical parametric maps were calculated by a random-effects analysis. The analysis was restricted to the cortical surfaces and inter-subject normalization was performed by spherical (rather than volumetric) registration to the surface of the MNI standard brain (see above). Group significance maps were thresholded to *p* = 0.01. Additionally only clusters of contiguous vertices exceeding this threshold and spanning at least 120 mm^2^ (approximately 10 functional voxels along the cortical surface) were considered.

Our primary motivation for the group analysis was to identify sensory-specific and putative multisensory regions of interest. Therefore, our analysis focused on five major contrasts: brain areas engaged in combined auditory and visual processing ([ASVS + ABVB + ASVB + ABVS]/4 vs. A0V0), brain areas engaged in auditory processing ([ASV0 + ABV0]/2 vs. A0V0), brain areas associated with phonological processing (ASV0 vs. ABV0), brain areas engaged in visual processing ([A0VS + A0VB]/2 vs. A0V0), and brain areas associated with processing body movements (A0VB vs. A0VS). ROIs were defined based on the group-average cortical significance maps (thresholded to *p* = 0.001) of these five main contrasts. Unisensory contrasts were used to define brain areas that were assumed to be modality-specific. These included the auditory cortex, the visual cortex, and predominantly visual areas within the parietal or frontal cortex. Moreover, a phonological processing region in the lateral superior temporal gyrus (lSTG) (Turkeltaub and Coslett, 2010; Woods et al., 2011) was defined by comparing auditory speech and body sounds (ASV0 vs. ABV0). Similarly, an extrastriate body area (EBA) (Peelen and Downing, 2007; Taylor and Downing, 2011) was defined by comparing visual body and lip movements (A0VB vs. A0VS). Brain areas assumed to be involved in multisensory processing were defined by comparing bimodal and unimodal contrasts. In particular, msSTS was assumed to be a brain region within the STS that responded to bimodal as well as to unimodal stimuli. However, bimodal contrasts had a higher statistical power than unimodal contrasts (as there were twice as many conditions). Consequently, at a given threshold bimodal contrasts showed slightly larger activity maps in putative multisensory regions than unimodal contrasts. Therefore, the borders of putative msSTS were marked based on bimodal contrasts. Unimodal responses within msSTS were verified by unimodal activity maps with a reduced threshold (*p* = 0.05). Implications of this relatively liberal definition criterion on our main findings are considered in the discussion (see below). Group labels (ROIs based on the cortical reconstruction) were reverse-mapped to individual cortical surfaces based on the spherical registrations. Both functional and structural landmarks (gyri and sulci) were used to segregate neighboring labels (ROIs).

### DWI-based tractography

#### Pre-processing

DWI pre-processing and fiber tracking was conducted with the FDT toolbox of FSL (Centre for Functional Magnetic Resonance Imaging of the Brain, University of Oxford, Oxford, UK) (Smith et al., 2004; Woolrich et al., 2009). First, all diffusion-weighted runs were concatenated and corrected for head motion and image distortions due to eddy currents. Then, distributions on diffusion parameters were estimated for each voxel by means of Markov Chain Monte Carlo sampling (Behrens et al., 2003). Two fibers were estimated for each voxel unless prevented by automatic relevance detection (Behrens et al., 2007) in order to model complex fiber architectures. Finally, the first b-zero weighted image of the DWI series was automatically co-registered to the high-resolution T1-weighted anatomical image using Freesurfer tools. Each co-registration was inspected by “blink comparison” and manually corrected if necessary.

#### Fiber tracking

The fiber tracking procedure essentially followed the protocol as described elsewhere (Beer et al., 2011b). In brief, fibers were tracked by repetitively sampling from the distributions on voxel-wise principal diffusion directions. Each time a streamline through these local samples was calculated. Connectivity distributions were built by sampling many streamlines. We computed 5000 trajectories per seed voxel (resulting in 5000 × number of seed voxels tracks) with 2000 steps per sample (step length was 0.5 mm). Streamline trajectories were terminated when the angle between two steps fell below 60° (curvature threshold) or when the trajectory turned back on itself (loop criterion). Furthermore, tracking was constrained to the ipsilateral cortex. No FA threshold was applied. Path distributions were corrected for the length of the pathways. All trajectories were seeded with labels (ROIs) derived from the functional analysis (see above). These included the Heschl's region (H), the planum temporale (PT), msSTS, lSTG (sensitive to phonological sounds), and the EBA. Moreover, for further analysis several other labels (ROIs) based on the results of the functional or tractographic whole-brain group analysis were used as seeds (see “Results”). All labels (and seeds) were defined in structural space. Note that tracking was performed in diffusion space whereas tracking results were transformed back into structural space (same as seed space) using the co-registration matrices (see above).

The tracking analysis was limited to the ipsilateral cortical terminations of the tracks. This procedure proved to be most informative in previous studies (Beer et al., 2011b). For each voxel along the cortical surface, track probabilities were calculated by dividing the number of tracks reaching this voxel by the overall number of tracks. In order to reject (reduce) false positive tracks, probability maps were thresholded to *p* = 5 × 10^−4^. This threshold was adopted from our previous study (Beer et al., 2011b). Individual thresholded track termination maps were projected to the reconstructed surface of the standard brain by spherical surface-based (rather than volumetric) normalization (Fischl et al., 1999b) and aggregated. Finally, group aggregated track termination maps were thresholded (only track terminations found in at least 3 subjects were considered). Moreover, only track terminations that formed clusters spanning at least 120 mm^2^ on the cortical surface were considered.

Previous research has shown that tracks seeded in the auditory cortex terminated in two distinct regions of the STS: anterior (aSTS) and posterior (pSTS). As we were interested in their role in multisensory processing, two ROIs (labels) were defined based on the group aggregate track termination maps for tracks seeded in H and PT, respectively. Moreover, additional ROIs (labels) in the inferior occipital cortex (IOC) were defined based on the results of the whole-brain track termination analysis (described in the “Results” section).

### ROI analysis of fMRI

Pre-processing for the ROI analysis was identical to the whole-brain analysis except that no spatial smoothing was applied. The mean MR signal change for each ROI was extracted by the GLM similar to the whole-brain analysis. In particular, the mean MR signal was estimated for each of the eight stimulus conditions and the baseline condition (A0V0). In addition, target trials (10% beep or 10% red dot) were modeled by separate predictors in order to exclude possible response-related activity. BOLD signals were calculated by subtracting the baseline signal (A0V0) from the MR signal of all eight stimulus conditions (e.g., [ASVS] - [A0V0]). Moreover, all BOLD signals were normalized to percent signal change relative to the ROI-specific global mean (constant predictor of the GLM). Our analysis focused on two aspects: (1) BOLD signals in response to auditory, visual, and bimodal stimuli regardless of stimulus type (speech or body movements) and (2) BOLD signal differences between speech (S) and body (B) stimuli reflecting feature-specific activity. For the first analysis, BOLD signals to speech and body (S, B) stimuli were pooled. One-sample *t*-tests were performed on BOLD signals for unimodal auditory and visual stimulus conditions in order to classify ROIs as being primary auditory, visual, or multimodal. In order to detect non-linear multisensory interaction effects (superadditive or subadditive) BOLD signals to combined auditory-visual stimuli were compared to the sum of BOLD signals for unimodal stimulus conditions (e.g., [ASVS + ABVB]/2 vs. [ASV0 + ABV0]/2 + [A0VS + A0VB]/2). In order to detect multisensory congruency effects, BOLD signals to incongruent bimodal stimuli (iAV) were compared to BOLD signals of congruent (cAV) bimodal stimuli (e.g., [ASVB + ABVS]/2 vs. [ASVS + ABVB]/2). Note that all BOLD signals reflect differences to the baseline (A0V0) condition normalized to percent signal change (see above). For the second analysis, BOLD signals to body movements (B) were subtracted from BOLD signals to speech stimuli (S). The same comparisons were performed as for the feature-unspecific analysis. Note that for incongruent bimodal stimuli (ASVB, ABVS) the sign of the S-B difference of visual stimuli (B-S) is opposite of auditory stimuli (S-B). Therefore, responses to bimodal stimuli were compared to the sum of unimodal responses showing the same sign ([ASVS-ABVB] vs. [ASV0-ABV0] + [A0VS-A0VB] for congruent bimodal stimuli; [ASVB-ABVS] vs. [ASV0-ABV0] + [A0VB-A0VS] for incongruent stimuli).

### Structural connectivity between ROIs

White matter connections between ROIs as defined by the whole-brain analysis were estimated by additional trackings. Here, each ROI served as seed for separate trackings. Moreover, each ROI served as target area. In order to derive a measure of pair-wise connectivity strength, we counted the number of tracks emitting from the seed ROI and reaching the target ROI. Note that the number of tracks emitting from the seed is proportionate to the number of seed voxels. Moreover, the number of tracks terminating in the target ROI increases with the number of target voxels. In order to compensate for this dependency on ROI size, a normalized track connectivity index (TCI) was calculated by dividing the number of tracks by the product of the number of voxels in the seed and target ROI, respectively. All ROIs served as both seed and target ROI. The resulting two connectivity indices were averaged. Moreover, connectivity indices from left and right hemispheres were averaged. Finally, TCI values from the whole group were averaged.

## Results

### Behavioral responses

All participants successfully detected most of the target stimuli (>95% hits, <1% false alarms). Moreover, no significant differences in response times were observed between visual and auditory targets suggesting that both modalities were attended equally well.

### Whole-brain analysis of fMRI

**Figure 2:**
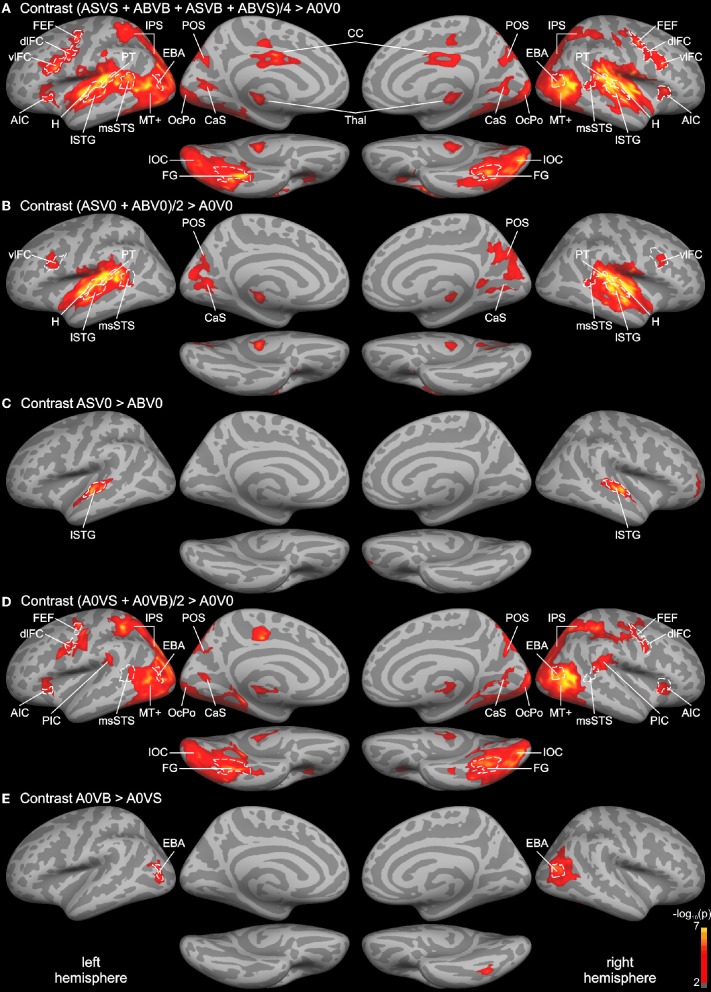
**Whole-brain group analysis statistical parametric maps overlaid on cortical surfaces of the MNI standard brain.** Both left and right hemispheres are shown from a lateral, medial, and inferior view. Five relevant contrasts are shown: **(A)** Contrasting BOLD responses to all bimodal stimuli with BOLD responses to control stimuli (A0V0) showed brain areas relevant for auditory, visual, and multisensory processing. **(B)** Brain areas primarily involved in auditory processing were identified by contrasting responses to unimodal auditory stimuli with the control. **(C)** Brain areas specific to phonological processing were identified by contrasting auditory phonological (lip) sounds (AS) with body sounds (AB). **(D)** Brain areas primarily involved in visual motion processing were identified by contrasting responses to unimodal visual stimuli with the control condition. **(E)** Brain areas specific to body processing were revealed by contrasting responses to visual body movements (VB) with visual lip movements (VS). All contrasts were thresholded to *p* = 0.01 (red) and color-coded (yellow: *p* = 10^−7^). An additional cluster threshold of 120 mm^2^ was applied. Regions of interests (ROIs) were defined as cortical labels (marked in white) based on functional (threshold *p* = 0.001) and structural (gyri and sulci) criteria (see text). Labels (ROIs) are indicated as dashed white lines. AIC, anterior insular cortex; CaS, calcarine sulcus; CC, cingulate cortex; CenS, central sulcus; dlFC and vlFC, dorsal and ventral parts of posterior lateral frontal cortex; EBA, extrastriate body area; FEF, frontal eye field; FG, fusiform gyrus; H, Heschl's region; IOC, inferior occipital cortex; IPS, intraparietal sulcus; lSTG, lateral superior temporal gyrus; msSTS, multisensory superior temporal sulcus; MT+, motion-sensitive middle temporal area plus satellites; OcPo, occipital pole; PIC, parieto-insular cortex; POS, parieto-occipital sulcus; PT, planum temporale.

In order to identify unisensory and putative multisensory brain regions involved in auditory and visual processing, a whole-brain analysis of the functional data was performed (see Figure 2). First, the response to all bimodal stimuli ([ASVS + ABVB + ASVB + ABVS]/4) was contrasted with the response to the control condition A0V0 (silence, blank screen). The BOLD response for this contrast revealed a large network of brain areas primarily in the temporal and occipital cortex extending to dorsal parietal and posterior frontal areas (Figure 2A). Comparing responses to unimodal auditory stimuli ([ASV0 + ABV0]/2) with the baseline condition (A0V0) showed that activity in the STC primarily reflected brain areas relevant for auditory processing (Figure 2B). Activity maps for both contrasts overlapped with the Heschl's region and the planum temporale—representing the core and caudal belt of auditory cortex, respectively (Petkov et al., 2006; Da Costa et al., 2011). The activity map in the auditory cortex further extended to lateral parts of the STG, which likely correspond to lateral belt and parabelt regions of the auditory cortex. The contrast comparing responses to speech sounds with sounds generated by body movements (AS vs. AB) (Figure 2C) revealed a relatively distinct region in the lateral STG associated with phonological processing (Turkeltaub and Coslett, 2010; Woods et al., 2011).

The contrast comparing responses to unimodal visual stimuli ([A0VS + A0VB]/2) with responses to the baseline condition (A0V0) revealed a network of brain areas primarily in the occipital and dorsal parietal cortex (Figure 2D). Two small clusters of activity were found in the anterior part of the calcarine sulcus (CaS) and the occipital pole (OcPo), respectively. These two clusters fell within the primary visual cortex (V1) as verified by the automatic parcellation of Freesurfer and likely reflect peripheral and central representations of the visual field. Larger clusters were found in the lateral occipital cortex stretching anterior to the posterior part of the middle temporal gyrus—a region overlapping with the motion-sensitive MT complex (MT+) (Zihl et al., 1983; Tootell et al., 1995). Activities in the parietal cortex were limited to ventral and dorsal parts of the intraparietal sulcus (IPS). A relatively large cluster of activity was found in the inferior and lateral parts of the occipito-temporal cortex. The posterior part of this cluster overlapped with track terminations (see below). The anterior part of this cluster was primarily found in the fusiform gyrus (FG). Furthermore, a small cluster of activity was found in the parietal (PIC) and the anterior insular cortex (AIC). Moreover, several adjacent clusters of activity were observed in ventral (vlFC) and dorsal (dlFC) parts of the posterior lateral frontal cortex. The contrast comparing responses to body movements with speech (lip) movements (VB vs. VS) revealed two relatively distinct regions in the lateral occipital cortex and FG presumably reflecting the EBA and the fusiform body area (Peelen and Downing, 2007; Taylor and Downing, 2011).

Although both unimodal auditory and unimodal visual contrasts showed distinct activity patterns throughout most of the cortex, several brain regions were activated by unimodal auditory, visual, and bimodal contrasts. This putative multisensory brain network included a region in the posterior part of the STS that most likely corresponds to the msSTS area (Beauchamp et al., 2004b, 2008). Note that the borders of msSTS were based on the bimodal contrast and verified by unimodal contrasts with a lower threshold (see “Materials and Methods” section for details). In addition, regions in the parieto-occipital sulcus (POS) and the CaS were activated by visual, auditory, and bimodal stimuli. Finally, the ventrolateral frontal cortex was responsive to both unimodal and bimodal stimuli. Note that most functional activities were similar in the two hemispheres.

### Whole-brain analysis of track terminations

**Figure 3:**
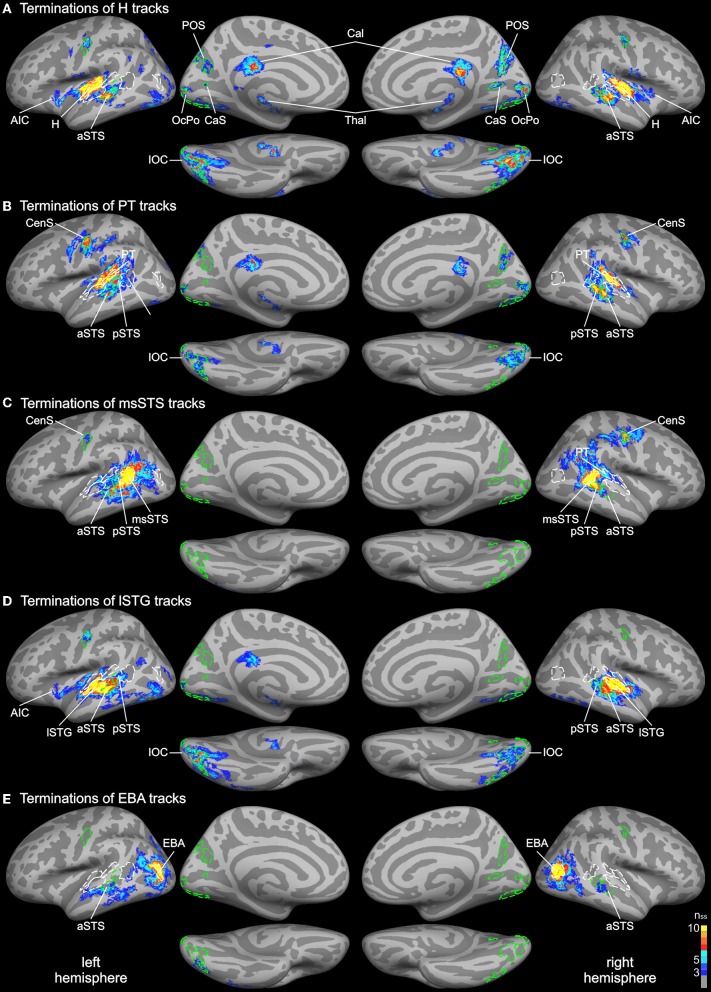
**Group average track termination maps overlaid on cortical surfaces of the MNI standard brain.** Termination maps were thresholded to *n*_ss_ = 3. Color scale: dark blue, terminations found in three hemispheres (threshold); yellow, terminations found in all hemispheres. The different panels show separate termination maps for tracks seeded in **(A)** Heschl's region (H), **(B)** planum temporale (PT), **(C)** multisensory STS (msSTS), **(D)** lateral STG (lSTG) sensitive to phonological sounds, **(E)** extrastriate body area (EBA). Labels (ROIs) based on track terminations are indicated as dashed green lines. Functional labels are shown in white. AIC, anterior insular cortex; aSTS and pSTS, anterior and posterior region of superior temporal sulcus; CaS, calcarine sulcus; CenS, central sulcus; IOC, inferior occipital cortex; OcPo, occipital pole; POS, parieto-occipital sulcus.

**Figure 4:**
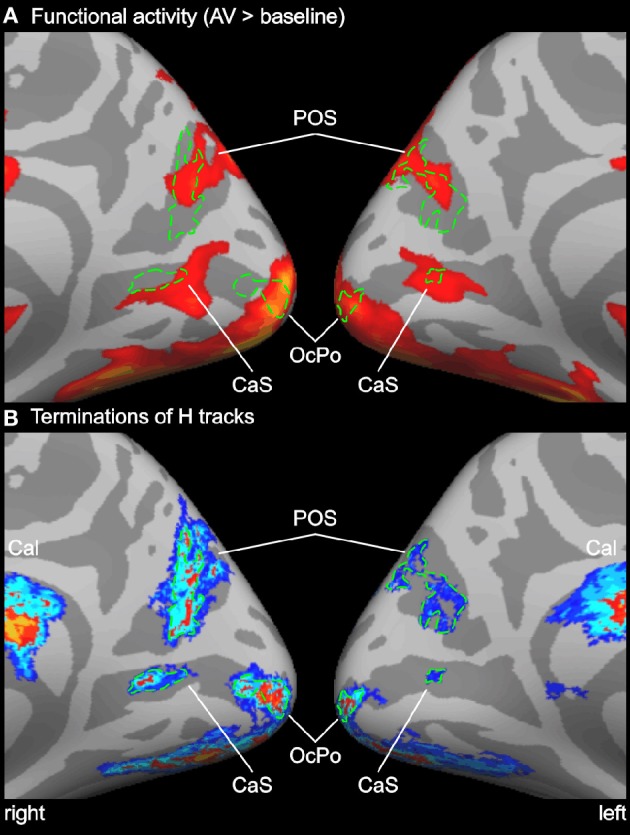
**Enlarged view of functional activity and track terminations in medial occipital cortex. (A)** Functional activity contrasting BOLD responses to all bimodal stimuli with BOLD responses to control stimuli (A0V0). **(B)** Termination maps for tracks seeded in the Heschl's region (H). Labels (ROIs) based on track terminations are shown in green. See also Figures 2 and 3 for abbreviations.

Probabilistic tracking was performed in the same participants to determine the white matter connectivity across cortical brain regions involved in auditory and visual processing of objects. The tracking algorithm was seeded in several auditory, visual, and multimodal cortical ROIs (labels) as determined by functional and structural (gyral and sulcal structure) criteria (see above). Figure 3 shows the cortical track terminations of the whole group for several seed regions. Only track terminations that exceeded the track frequency threshold in at least three individual brains and which exceeded the cluster threshold are displayed. Tracks seeded in the Heschl's region primarily terminated in several distinct cortical regions of the temporal and occipital cortex (Figure 3A). Track terminations in the temporal cortex were seen in adjacent auditory cortex including the planum temporale and the lSTG. Projections were also observed in an anterior division of the STS (aSTS). Furthermore, several foci of H track terminations were observed in the IOC (see details below). H track terminations were also found in several areas of the medial occipital lobe (see Figure 4 for an enlarged view): the OcPo, a region in the anterior CaS, and dorsal POS. Other track terminations were found at the cortical border to the corpus callosum (Cal) and thalamus (Thal)—most likely reflecting inter-hemispheric and thalamic fiber connections—and the anterior insula.

**Figure 5:**
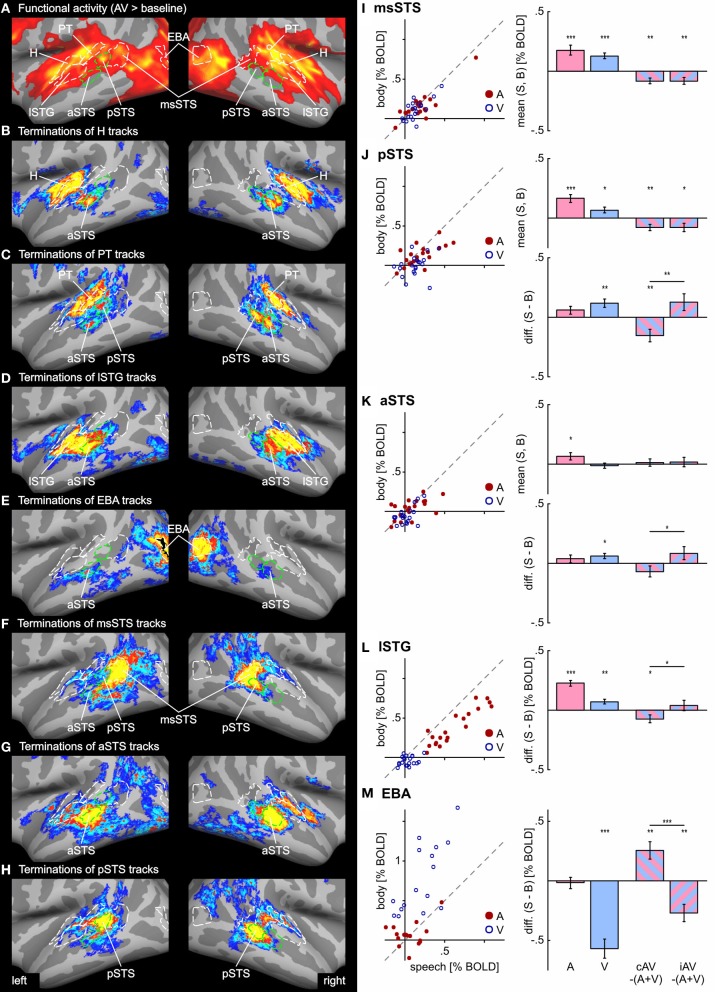
**Functional activity and track terminations in temporal cortex.** Enlarged views of functional activity and track termination maps are presented in panels **(A–H)**. Labels (ROIs) based on track terminations are shown in green, labels (ROIs) based on functional activity in white. Panels **(I–M)** present the results of the functional ROI analysis for temporal cortex regions (see Tables 1 and 2 for other ROIs). BOLD responses to unimodal (auditory or visual) conditions (relative to baseline) are shown separate for all hemispheres (*n* = 20) in scatter plots. Deviations from the main diagonal indicate specificity for stimulus type (speech or body). Bar graphs depict the group mean BOLD responses to unimodal conditions as well as differences between bimodal responses to the sum of unimodal responses (AV − [A + V]) separate for congruent (cAV) and incongruent (iAV) stimulus pairs (^***^p < 0.001; ^**^p < 0.01; ^*^p < 0.05). Separate graphs are shown for the mean responses across stimulus type and the response difference between speech and body (S-B) stimuli. See also Figures 2 and 3 for abbreviations.

Combined functional and structural criteria were adopted for the PT seed. Only the part of the planum temporale that was functionally active during the auditory or bimodal task was included. Tracks seeded in PT terminated in two distinct regions of the STS: anterior (aSTS) and posterior (pSTS) divisions (see Figure 5 for an enlarged view). PT tracks further projected to the IOC and a distinct region in the central sulcus (CenS). Tracks also reached the hemisphere border to the corpus callosum. Little or no PT track terminations were observed in the medial occipital lobe.

Our primary interest was in the connectivity profile of the msSTS region. Therefore, tracks were seeded in the part of the STS that was active during the multisensory localizer task (see above). Our primary interest was to evaluate the white matter connectivity of this region with auditory and visual cortex. As illustrated in Figure 3C, little or no connections between the msSTS region and the Heschl's region of the auditory cortex or early visual cortex (e.g., medial occipital cortex) were observed. Moreover, no track terminations were observed in the IOC. Instead terminations of the msSTS region primarily terminated in the planum temporale of the auditory cortex, the lateral STG, other parts of the STS, the middle temporal gyrus, inferior parietal cortex, and the CenS.

Our functional analysis revealed two regions that were either specific to phonological processing (lSTG) or visual body movements (EBA). Both regions showed signs of multisensory interactions (see below). Therefore, we were interested in the connectivity profile of these two regions. Tracks seeded in the phonological processing area (lSTG) showed wide-spread terminations in the auditory cortex including the H and PT region and the STS (Figure 3D). However, STS terminations were primarily observed in the aSTS region with limited connections to the msSTS region. Substantial track terminations were observed in the IOC. Tracks seeded in the EBA (Figure 3E) primarily terminated in adjacent regions. In addition, white matter connections were observed with the aSTS region in both hemispheres in some brains.

**Figure 6:**
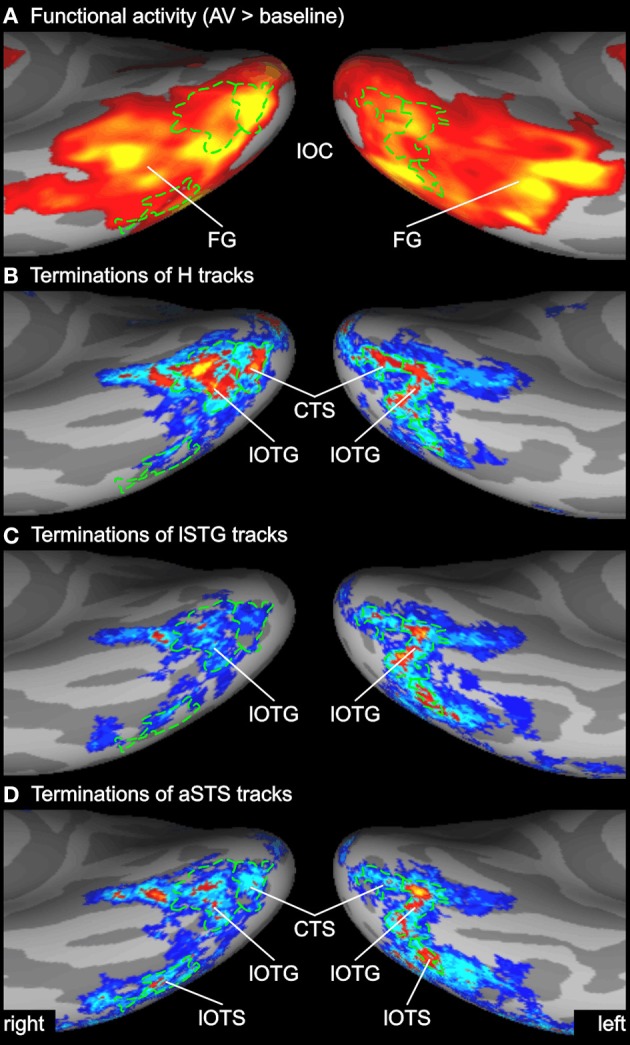
**Enlarged view of functional activity and track terminations in inferior occipito-temporal cortex. (A)** Functional activity contrasting BOLD responses to all bimodal stimuli with BOLD responses to control stimuli (A0V0). **(B–D)** Termination maps for tracks seeded in the Heschl's region (H), lateral superior temporal gyrus (lSTG), and anterior superior temporal sulcus (aSTS), respectively. Labels (ROIs) based on track terminations are shown in green. CTS, collateral transverse sulcus; lOTG and lOTS, lateral occipito-temporal gyrus and sulcus. See also Figures 2 and 3 for other abbreviations.

In order to compare functional activity maps with track termination maps enlarged views of the cortical surfaces are shown in Figures 4–6. As shown in Figure 4, H track terminations in the medial occipital cortex (CaS, OcPo, POS) corresponded quite well with the activity clusters as determined by the functional localizer. However, the connectivity profile of tracks terminating in the STS (Figure 5) was more complex than expected. One of the most relevant findings was that H and PT track terminations (aSTS and pSTS) showed little or no overlap with the functional msSTS region. In order to further investigate the connectivity profile of the STS, we performed additional tracking using the aSTS and pSTS regions as seeds. The results of this tracking revealed that the aSTS region showed relatively strong connections to the auditory cortex (H and PT), the phonological processing area (lSTG) as well as to the msSTS region. By contrast, the pSTS region was primarily connected to posterior parts of the auditory cortex (PT) and the aSTS region. These findings suggest that the msSTS region showed no direct white matter connections with the primary auditory cortex (H) but instead is connected to the auditory cortex via intermediate brain areas such as PT and aSTS.

Several previous studies have shown that activity in the STS is accompanied by activity in the inferior or lateral occipital cortex (Beauchamp et al., 2004b; Meyer et al., 2011). Figure 6 compares the activity maps of our functional localizer with the connectivity profiles of tracks terminating in the IOC. Comparing terminations of tracks from different seeds suggests that the IOC can be sub-divided into three main areas based on their connectivity profile. At the most posterior end—overlapping with the collateral transverse sulcus (CTS)—terminations were primarily found from H tracks. Adjacent to the CTS—overlapping with posterior parts of the lateral occipito-temporal gyrus or FG (lOTG)—terminations were observed from H tracks, lSTG tracks, and aSTS tracks. More lateral—overlapping with the lateral occipito-temporal sulcus (lOTS)—track terminations were primarily observed from lSTG tracks and aSTS tracks. Note that no direct white matter connections were observed between the STC and anterior parts of the lateral occipito-temporal/FG that showed activity in the fMRI analysis (but see ROI connectivity analysis below).

### ROI analysis of fMRI

In order to further quantify the contribution of each ROI on multisensory processing, we performed a ROI analysis on the functional data. We were primarily interested in determining the extent to which the region was primarily auditory, visual, or multisensory. Therefore, we compared the mean activity of each ROI during unimodal auditory (ASV0, ABV0) or visual stimulation (A0VS, A0VB) with the baseline condition (A0V0) (see Table 1 and Figures 5I–M). Furthermore, we were interested in whether the response to bimodal stimuli was larger (superadditive) or smaller (subadditive) than the sum of unimodal responses. Therefore, the sum of unimodal responses was subtracted from the response of congruent (ASVS, ABVB) and incongruent (ASVB, ABVS) stimuli, respectively. Finally, responses to congruent and incongruent were compared. Repeated-measures ANOVAs conducted separately for each comparison showed that BOLD responses significantly differed across ROIs. Table 1 shows the results of the ROI analysis averaged across speech and body movement conditions. As expected ROIs in the auditory cortex (H, PT) showed significant BOLD responses to auditory but not visual stimuli. Furthermore, unimodal auditory stimuli elicited significant BOLD responses in the lateral STG and the STS. Interestingly, auditory stimuli also elicited BOLD responses in several brain areas that were assumed to be primarily visual: CTS, lOTG, POS, CaS, and OcPo. Unimodal visual responses were observed in the medial occipital cortex (OcPo, POS) and the inferior occipito-temporal cortex (CTS, lOTS, lOTG, FG). Furthermore, visual responses were observed in the EBA region and posterior parts of the STS (pSTS, msSTS). No significant unimodal visual responses were observed in the auditory cortex.

**Table 1:** **Mean BOLD responses per ROI**.

**Label**	**Unimodal**				**Bimodal**					
	**A**		**V**		**cAV−(A+V)**		**iAV−(A+V)**		**iAV−cAV**	
	**Mean**	***(SE)***	**Mean**	***(SE)***	**Mean**	***(SE)***	**Mean**	***(SE)***	**Mean**	***(SE)***
H	1.06^***^	*(0.06)*	0.02	*(0.01)*	−0.05	*(0.03)*	−0.04	*(0.03)*	0.01	*(0.01)*
PT	0.73^***^	*(0.06)*	0.03	*(0.02)*	−0.06^*^	*(0.03)*	−0.01	*(0.04)*	0.05^*^	*(0.02)*
lSTG	0.51^***^	*(0.05)*	0.00	*(0.01)*	−0.01	*(0.03)*	−0.01	*(0.03)*	0.00	*(0.01)*
aSTS	0.06^*^	*(0.03)*	−0.02	*(0.02)*	0.02	*(0.04)*	0.01	*(0.03)*	−0.01	*(0.02)*
pSTS	0.16^***^	*(0.03)*	0.07^*^	*(0.02)*	−0.08^*^	*(0.03)*	−0.08^**^	*(0.03)*	0.00	*(0.02)*
msSTS	0.17^***^	*(0.04)*	0.13^***^	*(0.03)*	−0.08^**^	*(0.03)*	−0.09^**^	*(0.02)*	0.00	*(0.01)*
EBA	0.07	*(0.03)*	0.49^***^	*(0.08)*	−0.13^*^	*(0.04)*	−0.04	*(0.03)*	0.09^*^	*(0.04)*
lOTG	0.07^*^	*(0.03)*	0.38^***^	*(0.05)*	−0.07^*^	*(0.03)*	−0.03	*(0.02)*	0.04	*(0.02)*
lOTS	0.01	*(0.03)*	0.18^***^	*(0.03)*	−0.04	*(0.04)*	0.02	*(0.03)*	0.05^*^	*(0.02)*
CTS	0.11^**^	*(0.03)*	0.47^***^	*(0.05)*	−0.09^*^	*(0.04)*	−0.03	*(0.03)*	0.06^*^	*(0.03)*
FG	0.06	*(0.05)*	0.31^***^	*(0.04)*	−0.12^*^	*(0.05)*	−0.05	*(0.04)*	0.07	*(0.03)*
POS	0.10^**^	*(0.03)*	0.10^**^	*(0.03)*	−0.10^*^	*(0.04)*	−0.05	*(0.03)*	0.06	*(0.03)*
CaS	0.15^**^	*(0.04)*	0.08	*(0.05)*	−0.07	*(0.03)*	−0.04	*(0.04)*	0.03	*(0.04)*
OcPo	0.11^**^	*(0.04)*	0.24^***^	*(0.05)*	−0.10^**^	*(0.03)*	−0.03	*(0.04)*	0.07^*^	*(0.03)*
AIC	0.03	*(0.02)*	0.09^**^	*(0.02)*	−0.04	*(0.02)*	0.02	*(0.03)*	0.06^*^	*(0.02)*
CenS	0.06	*(0.03)*	0.06^**^	*(0.02)*	−0.09^*^	*(0.04)*	−0.05	*(0.04)*	0.04	*(0.02)*
dlFC	−0.02	*(0.03)*	0.14^***^	*(0.03)*	0.01	*(0.04)*	−0.01	*(0.03)*	−0.01	*(0.03)*
vlFC	0.07	*(0.04)*	0.11^**^	*(0.02)*	−0.05	*(0.02)*	−0.01	*(0.04)*	0.04	*(0.03)*

Note: The table lists the mean BOLD responses (percent signal change) relative to baseline (A0V0) averaged across speech (S) and body (B) conditions for each ROI (label). Standard errors (SE) are shown in parenthesis. Significant differences are highlighted in bold (

*** p < 0.001;

** p < 0.01;

* p < 0.05,

uncorrected). A, unimodal auditory; V, unimodal visual; cAV, congruent bimodal; iAV, incongruent bimodal. ROIs were defined as labels on cortical surfaces (see Figures 2, 3, 6). AIC, anterior insular cortex; aSTS and pSTS, anterior and posterior region of superior temporal sulcus; CaS, calcarine sulcus; CenS, central sulcus; CTS, collateral transverse sulcus; dlFC and vlFC, dorsal and ventral parts of the posterior lateral frontal cortex; EBA, extrastriate body area; FG, fusiform gyrus; H, Heschl's region; lOTG and lOTS, lateral occipito-temporal gyrus and sulcus; lSTG, lateral superior temporal gyrus; msSTS, multisensory superior temporal sulcus; OcPo, occipital pole; POS, parieto-occipital sulcus; PT, planum temporale.

Subadditive responses to bimodal stimuli (irrespective of stimulus type) were primarily observed in the posterior part of the STS (pSTS, msSTS). Moreover, subadditive responses to congruent bimodal stimuli were observed in the inferior occipito-temporal cortex (FG, lOTG, CTS), parts of the occipital cortex (POS, OcPo), and the planum temporale. Significant BOLD response differences between congruent and incongruent bimodal stimuli were observed in the planum temporale, EBA, IOC (lOTS, CTS), the OcPo, and anterior insula.

As our whole-brain analysis revealed cortical regions that responded preferably to speech sounds (S) or visual body movements (B), an additional ROI analysis was performed on the differences between S and B stimulus types (S-B) (see Table 2 and Figures 5J–M). Note that positive differences reflect stronger BOLD responses to speech (S) stimuli and negative differences reflect stronger responses to body (B) stimuli. Further note that responses to bimodal stimuli were compared with the sum of its corresponding unimodal differences (see “Methods and Materials” section). Repeated-measures ANOVAs conducted separately for each comparison showed that BOLD responses significantly differed across ROIs. As expected from the results of the whole-brain analysis, the lSTG region showed stronger responses to unimodal phonological sounds (ASV0) than to body sounds (ABV0). By contrast, weaker responses to speech sounds were observed in the OcPo. Similarly, the EBA region and the anterior part of the FG showed stronger responses to body movements (A0VB) than to lip (speech) movements (A0VS). In addition, unimodal visual lip (speech) movements (A0VS) elicited stronger responses than body movements (A0VB) in the phonological STG region (lSTG), subparts of the STS (aSTS and pSTS but not msSTS), the inferior (CTS) and posterior (OcPo) occipital cortex.

**Table 2:** **Difference (speech minus body) BOLD responses per ROI**.

**Label**	**Unimodal**				**Bimodal**					
	**A**_**S−B**_		**V**_**S−B**_		**cA**_**S−B**_**V**_**S−B**_**−(A**_**S−B**_**+V**_**S−B**_**)**		**iA**_**S−B**_**V**_**B−S**_**−(A**_**S−B**_**−V**_**S−B**_**)**		**(iAV−[A−V]**_**i**_**)−(cAV−[A+V]**_**c**_**)**	
	**Mean**	***(SE)***	**Mean**	***(SE)***	**Mean**	***(SE)***	**Mean**	***(SE)***	**Mean**	***(SE)***
H	0.03	*(0.04)*	0.03	*(0.02)*	−0.01	*(0.04)*	0.00	*(0.07)*	0.01	*(0.06)*
PT	0.03	*(0.04)*	0.02	*(0.03)*	−0.03	*(0.05)*	−0.07	*(0.06)*	−0.04	*(0.05)*
lSTG	0.22^***^	*(0.02)*	0.07^**^	*(0.02)*	−0.08^*^	*(0.03)*	0.04	*(0.04)*	0.11^*^	*(0.05)*
aSTS	0.04	*(0.03)*	0.06^*^	*(0.02)*	−0.07	*(0.05)*	0.08	*(0.05)*	0.15^*^	*(0.07)*
pSTS	0.06	*(0.03)*	0.12^**^	*(0.04)*	−0.16^**^	*(0.05)*	0.12	*(0.07)*	0.28^**^	*(0.09)*
msSTS	0.03	*(0.02)*	0.02	*(0.02)*	−0.09	*(0.05)*	0.01	*(0.05)*	0.10	*(0.07)*
EBA	−0.02	*(0.05)*	−0.57^***^	*(0.08)*	0.25^**^	*(0.07)*	−0.27^**^	*(0.07)*	−0.53^***^	*(0.11)*
lOTG	−0.02	*(0.03)*	0.07	*(0.04)*	0.00	*(0.06)*	0.06	*(0.05)*	0.06	*(0.06)*
lOTS	−0.07	*(0.05)*	−0.03	*(0.03)*	0.01	*(0.05)*	0.04	*(0.06)*	0.03	*(0.07)*
CTS	−0.04	*(0.04)*	0.11^*^	*(0.04)*	0.02	*(0.08)*	0.14	*(0.07)*	0.12	*(0.08)*
FG	0.05	*(0.06)*	−0.07^*^	*(0.03)*	−0.05	*(0.07)*	−0.05	*(0.11)*	−0.01	*(0.09)*
POS	0.01	*(0.03)*	−0.03	*(0.03)*	−0.02	*(0.05)*	−0.04	*(0.05)*	−0.01	*(0.05)*
CaS	−0.02	*(0.05)*	−0.02	*(0.07)*	0.03	*(0.12)*	−0.01	*(0.07)*	−0.04	*(0.15)*
OcPo	−0.10^***^	*(0.03)*	0.16^**^	*(0.05)*	0.06	*(0.06)*	0.13^*^	*(0.05)*	0.07	*(0.08)*
AIC	0.02	*(0.03)*	−0.03	*(0.03)*	−0.05	*(0.05)*	−0.04	*(0.05)*	0.01	*(0.06)*
CenS	−0.01	*(0.03)*	−0.02	*(0.03)*	0.02	*(0.04)*	0.02	*(0.06)*	0.00	*(0.07)*
dlFC	0.09^*^	*(0.04)*	0.05	*(0.06)*	−0.16	*(0.08)*	0.05	*(0.10)*	0.22	*(0.14)*
vlFC	0.03	*(0.04)*	0.05	*(0.03)*	−0.21^***^	*(0.04)*	0.03	*(0.08)*	0.24^*^	*(0.09)*

Note: The table lists the differences in BOLD responses (percent signal change) to speech (S) minus body (B) stimuli for each ROI (label). Standard errors (SE) are shown in parenthesis. Significant differences are highlighted in bold (

*** p < 0.001;

** p < 0.01;

* p < 0.05,

uncorrected). A_S−B_, unimodal auditory; V_S−B_, unimodal visual; cA_S−B_V_S−B_, congruent bimodal; iA_S−B_V_B−S_, incongruent bimodal. See also Table 1 for abbreviations.

Congruent bimodal stimuli elicited subadditive difference responses (ASVS-ABVB) in several brain regions including lSTG, pSTS (but not msSTS), and the EBA. By contrast, incongruent bimodal stimuli (ASVB-ABVS) elicited superadditive responses compared to their unimodal counter-parts in most of these regions including the OcPo. Accordingly, a significant difference in the bimodal response pattern of congruent and incongruent bimodal stimuli was observed in lSTG, aSTS, pSTS, EBA, and vlFC, suggesting that congruent and incongruent bimodal stimuli were processed differently in these areas.

### Structural connectivity between ROIs

Our ROI analysis on functional data and our whole-brain tracking results indicated that several distinct brain areas were involved in the processing of auditory and visual stimuli. Therefore, we performed pair-wise probabilistic tracking in order to quantify the strengths of white matter connections using the ROIs as seed and targets. Track probabilities were normalized by the size of the seed and target regions, respectively. The group average normalized ROI-to-ROI track probabilities (pooled across both hemispheres) are shown in Table 3. This analysis primarily confirmed the results on the whole-brain track terminations reported above. That is, H tracks primarily terminated in the lSTG, aSTS (but not msSTS), IOC, and medial occipital cortex. PT tracks primarily terminated in the lSTG, aSTS, pSTS, CenS, and dlFC. Tracks seeded in the phonological lSTG area primarily terminated in the aSTS and pSTS region as well as the lateral inferior occipito-temporal cortex. The aSTS region showed a connectivity profile distinct from the more posterior pSTS and msSTS areas. Whereas the aSTS region showed strong projections to the IOC, no such projections were observed in more posterior regions of the STS (pSTS, msSTS). The pSTS region showed relatively strong connectivity with area PT. The msSTS region was primarily connected with the aSTS region. The EBA was primarily connected with aSTS and sub-regions of the IOC (lOTS).

**Table 3:** **ROI-to-ROI white-matter connectivity**.

**Label**	**PT**	**lSTG**	**aSTS**	**pSTS**	**msSTS**	**EBA**	**lOTG**	**lOTS**	**CTS**	**FG**	**POS**	**CaS**	**OcPo**	**AIC**	**CenS**	**dlFC**	**vlFC**
H	**3.3**	**12.7**	**10.3**	**2.4**	1.1	0.4	**22.1**	**13.2**	**13.3**	0.6	**2.3**	1.3	**5.6**	<0.1	1.8	**2.3**	0.2
PT		**7.4**	**7.4**	**16.4**	**3.1**	0.2	**2.4**	**2.3**	**3.6**	<0.1	0.8	0.1	1.9	<0.1	**21.5**	**11.6**	0.2
lSTG			**40.4**	**5.9**	**2.4**	0.7	**8.0**	**17.7**	**4.4**	0.5	0.4	<0.1	1.7	<0.1	1.6	**3.2**	<0.1
aSTS				**6.5**	**8.0**	**2.7**	**24.7**	**35.3**	**8.1**	0.3	0.7	0.1	**5.5**	<0.1	**8.7**	**4.1**	<0.1
pSTS					**5.5**	0.2	0.7	0.2	0.5	<0.1	<0.1	<0.1	0.1	<0.1	**7.5**	0.6	<0.1
msSTS						**2.0**	0.2	0.2	0.3	<0.1	<0.1	<0.1	0.3	<0.1	<1.1	0.1	<0.1
EBA							0.4	**2.4**	<0.1	<0.1	0.1	<0.1	<0.1	<0.1	0.3	<0.1	<0.1
lOTG								**16.0**	**5.3**	0.7	0.4	**5.4**	1.5	**2.3**	0.1	<0.1	<0.1
lOTS									0.1	1.4	0.1	<0.1	0.2	**4.4**	<0.1	<0.1	<0.1
CTS										<0.1	1.0	**11.6**	**9.4**	0.4	<0.1	<0.1	<0.1
FG											<0.1	<0.1	<0.1	**3.2**	<0.1	<0.1	0.1
POS												**19.3**	0.9	0.1	0.1	<0.1	<0.1
CaS													**9.5**	<0.1	<0.1	<0.1	<0.1
OcPo														0.1	0.1	<0.1	<0.1
AIC															<0.1	<0.1	0.1
CenS																**14.0**	0.1
dlFC																	**7.2**

Note: The table lists group average ROI-to-ROI white-matter track connectivity indices (TCI) reflecting normalized track probabilities. The number of tracks connecting ROIs were divided by the product of voxels of each ROI. TCI-values are given in 10^−8^. Large values reflect high probabilities of tracks between the voxels of each ROI. Values greater than 2 are highlighted in bold. See also Table 1 for abbreviations.

The ROI-to-ROI connectivity analysis further revealed the connectivity profile of inferior occipital regions. These regions were primarily connected with the H part rather than the PT part of the auditory cortex. Furthermore, anterior regions of the IOC (lOTG, lOTS) were connected with the lSTG and the aSTS, but little connectivity was observed with the msSTS. Moreover, parts of the IOC (lOTG) were connected to the medial occipital cortex (CaS).

The ventrolateral frontal cortex as well as the anterior insular region observed in fMRI showed little direct connectivity with the STC. Instead these two regions were connected via inter-mediate nodes such as the dorsolateral frontal cortex or the inferior occipito-temporal cortex (e.g., FG, lOTS).

## Discussion

Our whole-brain analysis revealed a large network of brain areas responding to auditory, visual, or multimodal stimuli. Processing of unimodal auditory stimuli (Figures 2B,C) primarily involved the STC, distinct parts of the medial occipital cortex, and the ventrolateral frontal cortex. Activity clusters in the temporal lobe included the Heschl's region and the planum temporale, which likely correspond to the core and caudal belt of auditory cortex, respectively (Petkov et al., 2006; Da Costa et al., 2011). Consistent with previous research (Turkeltaub and Coslett, 2010; Woods et al., 2011) a relatively distinct region in the lateral anterior STG sensitive to phonological (speech) processing was observed in both hemispheres.

Processing of unimodal visual stimuli involved a network of brain areas primarily in the occipital and dorsal parietal cortex (Figures 2D,E). This network largely corresponds to brain networks related to visual motion processing as described elsewhere (Kovacs et al., 2008; Beer et al., 2009). It included activity in the posterior part of the middle temporal gyrus—a region known as the motion-sensitive MT complex (MT+) (Zihl et al., 1983; Tootell et al., 1995). The visual network also involved distinct regions in the medial occipital cortex (CaS and POS) and inferior and lateral parts of the occipito-temporal cortex. Two distinct regions in the lateral occipital cortex and FG, respectively, were sensitive to visual body movements. Similar regions were described before as EBA and fusiform body area (Peelen and Downing, 2007; Taylor and Downing, 2011). Moreover, several adjacent clusters of activity were found in the frontal cortex.

Although auditory and visual processing was associated with distinct brain areas throughout most of the cortex, several cortical sites were activated by both auditory and visual stimuli. In particular, we observed a region in the posterior part of the STS that was activated by auditory, visual, and bimodal stimuli (Figures 2, 5). Moreover, auditory and visual activity was also observed in the medial occipital cortex (POS, CaS) and the ventrolateral frontal cortex. We subsequently discuss the activity and connectivity profiles of sub-parts of the auditory-visual brain network in detail.

### Medial occipital cortex

The medial occipital cortex showed two clusters in the anterior part of the CaS and the OcPo that were activated by auditory-visual stimuli. These two regions likely reflect peripheral and central representations of the primary visual cortex (V1), respectively (e.g., Beer et al., 2009). In addition, auditory-visual stimuli elicited activity in dorsal parts of the POS. Although these regions are considered modality-specific visual areas, they also showed BOLD responses to purely unimodal auditory and subadditive responses to bimodal stimulation (see Table 1 and Figure 2). This finding of sound-induced activity and crossmodal response modulation in visual cortex is in accordance with a number of EEG/MEG (McDonald et al., 2003; Raij et al., 2010), positron emission tomography (Weeks et al., 2000; Gougoux et al., 2005), and fMRI (Röder et al., 2002) studies. Traditionally, this crossmodal recruitment of visual cortex was attributed to feedback signals from multisensory association cortex (McDonald et al., 2003). However, several lines of research suggest that there are even direct connections between primary sensory cortices (Foxe and Schroeder, 2005). For instance, sounds presented prior to a (peripheral) visual target facilitated visual perception only when sounds and visual stimuli were spatially aligned but not when they were misaligned by as little as 6 degrees of visual angle (Beer et al., 2011a). The sharp spatial tuning of crossmodal facilitation suggests that it relies on brain structures with constrained receptive fields. Similarly, sounds facilitate visual perceptual learning only at visual field locations that were aligned with the sound source (Beer and Watanabe, 2009). MEG combined with source analysis revealed that sounds elicited responses in primary visual cortex at latencies (53 ms) that seem to be too early to be mediated by association cortex (Raij et al., 2010). Functional connectivity MRI showed that BOLD signals between early sensory cortices are highly correlated whereas limited correlation was observed in other brain regions (Eckert et al., 2008; Lewis and Noppeney, 2010; Werner and Noppeney, 2010a). Anatomical tracer studies reported direct axonal connections between auditory and early visual cortex in non-human primates (Falchier et al., 2002; Rockland and Ojima, 2003; Clavagnier et al., 2004; Bizley et al., 2007). Recently, we reported direct white matter tracts between the Heschl's region and the medial occipital cortex in humans (Beer et al., 2011b). The present tracking results with seeds in the auditory cortex (H or PT) essentially replicated our previous finding on an independent sample. White matter tracks seeded in the Heschl's region terminated in anterior parts of the CaS, the OcPo, and the dorsal part of the POS. In addition, the present findings showed (whole-brain and ROI analysis) for the first time that these track termination areas also correspond to sub-parts of the visual cortex that were recruited by auditory processing.

### Temporal cortex

Consistent with previous findings (Calvert et al., 2000; Beauchamp et al., 2004b, 2008; Baumann and Greenlee, 2007; Hein et al., 2007; Noesselt et al., 2007; Van Atteveldt et al., 2010; Werner and Noppeney, 2010b; Meyer et al., 2011; Nath and Beauchamp, 2012; Plank et al., 2012) our functional MRI results showed that several brain regions of the temporal cortex were involved in auditory and visual object and action processing. Unisensory auditory processing primarily recruited superior parts of the temporal cortex whereas unisensory visual processing primarily involved inferior parts. These networks overlapped at the posterior part of the STS. It likely corresponds to the msSTS region as described in previous studies (Beauchamp et al., 2004b, 2008). No overlap of unimodal activity maps was observed in other STS regions. As indicated in the introduction, brain imaging techniques such as fMRI, EEG, or MEG detect responses of large neural ensembles and overlapping unimodal activity maps may simply result from separate but interspersed neural populations (Laurienti et al., 2005). However, multisensory integration may be inferred, if the brain responses to bimodal stimuli are not a linear summation of brain responses to unimodal stimuli (AV ≠ A + V). Therefore, we performed a ROI analysis comparing BOLD signals to bimodal (AV) with the sum of unimodal responses (A + V). This ROI analysis showed subadditive responses within our putative msSTS region. Note, however, that limitations to the criterion of non-linearity (AV − [A + V]) as stated elsewhere (Gondan and Röder, 2006; Proctor and Meyer, 2011; Szameitat et al., 2011) must be considered. That is, responses to two trials are subtracted from responses to one trial. Accordingly, it may be argued that subadditivity merely results from double subtraction of BOLD responses that are common to all trials. However, we believe that this argument may not (or only partially) account for multisensory interaction effects in our study for several reasons: Common BOLD responses may reflect cognitive processes associated with the task (e.g., alertness or motor responses, etc.). However, these task-dependent responses were minimized in our paradigm as we used passive viewing/listening and the biological stimuli were task-irrelevant. Additionally, common BOLD responses may result from task-independent activity that is observed even in the resting brain (Gusnard and Raichle, 2001). Although it is disputed whether this resting state activity reflects a task-independent default brain state or just another task-specific activity (Morcom and Fletcher, 2007), it should be considered when examining non-linear interactions. Note that our paradigm contained baseline trials that were similar to the stimulus trials on most aspects (e.g., timing, task, etc.) except that they did not contain the target stimuli. Therefore, these baseline trials likely elicited task-dependent and task-independent BOLD responses that are common to all trials. Further note that in our study the MR signal for this baseline (A0V0) was subtracted from the MR signal of each stimulus condition (e.g., ASVS). Therefore, common BOLD responses were likely eliminated by this comparison prior to testing for interaction effects. Furthermore, unspecific BOLD responses should affect all or most brain areas in a similar manner. However, we found no subadditive responses in several low-level and high-level brain areas such as primary auditory cortex (H), some parts of visual cortex (CaS), and frontal cortex (see Tables 1, 2). Furthermore, several brain areas (e.g., aSTS) also showed differential responses to congruent and incongruent bimodal stimuli. Congruency effects cannot be explained by double subtraction of common BOLD response components. Similarly, subadditive responses were also found to be selective for stimulus type (speech vs. body). These comparisons (A_S−B_V_S−B_ − [A_S−B_ + V_S−B_]) implicitly controlled for common response components. Another concern is whether multisensory interactions reflect perceptual or post-perceptual processing (McDonald et al., 2000). Our task (passive viewing) discouraged participants to adopt post-perceptual (e.g., decision, response, etc.) processes on the biological stimuli. However, observers may have engaged in such processes implicitly. If so, multisensory processing of biological stimuli should have interfered with the main (unimodal) detection task. No interference effects were observed.

Our finding of subadditive responses within the msSTS region is consistent with a number of EEG/MEG studies (Raij et al., 2000; Cappe et al., 2010) and electrophysiology studies in animals (Barraclough et al., 2005; Dahl et al., 2009) that observed similar subadditive responses in the STS. However, the nature of subadditive responses is still debated (Laurienti et al., 2005; Stein et al., 2009; Cappe et al., 2010). As subadditive responses in the superior colliculus were usually observed when auditory and visual stimuli were slightly mis-aligned, some researchers suggested that it reflects integration at the inhibitory surround receptive field of multisensory neurons (Stein and Stanford, 2008). However, subadditive responses in our and other studies (e.g., Barraclough et al., 2005) were observed even with spatially aligned auditory-visual stimuli. Another possibility is that crossmodal signals sharpen the tuning curve of object-encoding neurons (Raij et al., 2000). Alternatively, subadditive multisensory interactions may reflect converging auditory and visual input to the same neural (multimodal) representation of an object. As with salient stimuli—but not with degraded ones (Werner and Noppeney, 2010b)—either modality is sufficient to activate this representation, the response to bimodal stimuli reflects the maximum of unimodal responses (rather than their sum). Accordingly, subadditivity might reflect adaptation or saturation of a bimodal neural population (Weigelt et al., 2008) and its associated hemodynamic response (Toyoda et al., 2008).

Consistent with previous research (Meyer et al., 2011), most of the brain areas in the temporal cortex were recruited by both speech and body stimuli. However, a sub-region of that network (lSTG) showed additional selectivity for phonological sounds (compared to body action sounds). A similar region responsive to sub-lexical speech sounds was observed before (Turkeltaub and Coslett, 2010). It likely corresponds to the lateral belt or parabelt of the auditory cortex (Woods et al., 2011). Although the lSTG is primarily auditory, it was also activated by visual stimuli and showed subadditive responses to congruent bimodal stimuli. Consistent with our finding, intracranial recordings from the lateral belt of rhesus monkeys showed multisensory modulation of facial and vocal signals (Ghazanfar et al., 2005). Furthermore, a region in the lateral occipital cortex showed stronger responses to visual body movements compared to lip movements. This region most likely corresponds to the EBA as described before (Peelen and Downing, 2007; Cziraki et al., 2010; Taylor and Downing, 2011). Our ROI analysis showed that the BOLD response in the EBA was modulated by concurrent auditory stimuli. An enhanced response (superadditive) was observed with incongruent bimodal stimuli and a reduced response (subadditive) was observed for congruent bimodal stimuli. Previous research has shown that sounds can affect visual processing of biological motion (Baart and Vroomen, 2010). However, to our knowledge this is the first demonstration of superadditive response enhancement by concurrent auditory-visual stimuli in the EBA. Interestingly, sounds modulated the response to visual stimuli although sounds alone did not elicit responses in the EBA. However, as shown by previous animal physiology, even subthreshold auditory connections can substantially influence visual processing (Clemo et al., 2008).

The primary motivation for our study was to examine the structural connectivity of the multisensory integration regions in the STC with the auditory cortex and other relevant brain areas. Our previous study demonstrated white matter tracts between auditory cortex and two distinct regions within the STS (aSTS, pSTS). The tracking results of the present study essentially replicated these previous findings by showing that tracks seeded in the Heschl's region and the planum temporale terminated in an anterior (aSTS) and posterior (pSTS) part of the STS. We were interested in the relationship of these structurally-defined regions with the msSTS region that was observed with functional MRI (Beauchamp et al., 2004b, 2008). We expected that the functionally-defined msSTS region overlapped with the STS regions that were connected with the auditory cortex via white matter tracks. Contrary to this hypothesis we found only limited overlap suggesting that msSTS is not directly connected with the core (H) of the auditory cortex. Additional tracking revealed that the msSTS also showed little direct connectivity with early visual brain areas including IOC. Instead msSTS seems to be primarily connected to other STS regions such as aSTS. Note that the borders of our msSTS region were defined by relatively liberal criteria. Therefore, it is unlikely that our msSTS region was too small to show sufficient overlap with terminations from auditory cortex tracks. Our tracking results further showed that areas lSTG and EBA—both regions selective for stimulus type (S vs. B) and modulated by sensory signals from its non-preferred modality—showed no direct white matter connections. Instead these two regions seemed to be connected via intermediate nodes such as aSTS or the IOC (e.g., lOTS).

Our finding of no direct connections between msSTS and auditory cortex seem to be inconsistent with previous connectivity research. For instance, functional connectivity based on fMRI suggested direct connections between primary auditory or visual cortex with msSTS (Noesselt et al., 2007; Werner and Noppeney, 2010a). However, functional connectivity does not necessarily require direct (monosynaptic) anatomical connections but instead may be mediated by polysynaptic connections (Damoiseaux and Greicius, 2009). Our results are partially consistent with tracer studies in animals. For instance, retrograde tracers injected into the STS of rhesus monkeys revealed that separate parts of the STS receive afferents from segregated areas of the STG (Seltzer and Pandya, 1994). Similarly, we found at least two regions within the STS (aSTS and pSTS) that were connected to separate regions of the STG and auditory cortex. However, direct axonal connections were also observed between polysensory STS and V1 in monkeys (Falchier et al., 2002). By contrast, we did not observe corresponding white matter tracks in humans. Tracer studies in rodents also observed sparse axonal projections from auditory cortex to other parts of the brain that were not detected in our study (Budinger and Scheich, 2009). Some of these differences might reflect species-specific characteristics. Alternatively, these differences may result from methodological limitations of fiber tracking (see below). For instance, DWI-based fiber tracking tends to neglect small fibers. In addition, small fiber tracts may have been obscured by our clustering procedure that we adopted in order to minimize false positives.

Our tracking results suggest that multisensory integration in the STS is not mediated by a single brain area but instead by a cascade of inter-connected brain areas located in the lateral temporal cortex (and IOC). Our ROI analysis of functional MRI data further suggests that aSTS, pSTS, and msSTS differ by the pattern of multisensory processing. Whereas activity in the aSTS region was sensitive to stimulus type (speech vs. body), no such sensitivity was observed in msSTS. Moreover, some regions (e.g., aSTS, lSTG) were predominantly involved in auditory processing, but auditory responses were modulated by visual signals. Both our connectivity findings and our functional results suggest that the STC is subdivided into several distinct regions and is best conceived as a multisensory network or complex rather than as a single region. This notion of a multisensory STC network may also help us to understand conventional fMRI findings that are difficult to accommodate with the notion of a single msSTS region. For instance, several brain imaging studies in humans found multiple distinct brain areas within the STS that may be segregated based on their multisensory integration patterns (Beauchamp et al., 2004a; Van Atteveldt et al., 2010; Werner and Noppeney, 2010b; Stevenson et al., 2011; Noesselt et al., 2012). Recent electrophysiological recordings from the STS of rhesus monkeys revealed separate patches within the STS that differ by the type of multisensory interactions (e.g., superadditive vs. subadditive) (Dahl et al., 2009). Our results elaborate these reports by showing that STS patches related to multisensory processing may also be characterized by distinct “connectivity fingerprints” (Behrens and Sporns, 2012).

### Inferior occipito-temporal cortex

Our whole-brain analysis showed that visual speech and body motion also activated a relatively large cluster in the inferior and lateral occipito-temporal cortex. Our tracking results showed that the posterior part overlapped with track terminations from auditory cortex or the STC (H, lSTG, aSTS) (Figure 6). These track termination patterns further suggest subdivisions within this area: Tracks seeded in the Heschl's region primarily terminated in the collateral-transverse sulcus (CTS), tracks seeded in lSTG primarily terminated in the lOTG, and tracks seeded in aSTS terminated in the lOTS. No track terminations were observed in anterior parts of the inferior occipito-temporal cortex (primarily overlapping with the FG). Although all of these regions were primarily visual, two regions (CTS, lOTG) also showed BOLD responses to auditory stimuli. Moreover, bimodal stimuli elicited subadditive responses in the IOC. Similar multisensory responses in the IOC were observed in previous studies (Beauchamp et al., 2004b; Hocking and Price, 2008). Tracer studies in ferrets showed axonal connections between the auditory core and area 20 (corresponding to ventral/inferior occipital cortex) (Bizley et al., 2007). Moreover, axonal connections were observed between ventral preoccipital regions and the STS in rhesus monkeys (Yeterian and Pandya, 2010). Given that our tracking failed to find direct connections between STS areas and early visual cortex, it is possible that the IOC provides the major visual input to the STC regions.

### Frontal cortex

Several studies examining multisensory integration observed multimodal responses in the anterior insula and ventrolateral frontal cortex - in addition to STS activity (Calvert et al., 2000; Beauchamp et al., 2004b; Taylor et al., 2006; Hein et al., 2007; Meyer et al., 2011; Nath and Beauchamp, 2012). For instance, familiar incongruent stimuli (animal sounds paired with animal pictures)—but not pairs of unfamiliar artificial stimuli—elicited larger responses in the inferior frontal cortex compared to that evoked by congruent stimulus pairs (Hein et al., 2007). Electrophysiological recordings in non-human primates showed that the ventrolateral frontal cortex contains a relatively large proportion of bimodal neurons that are responsive to faces and animal vocalizations (Romanski, 2007). We also observed a brain area in the ventrolateral frontal cortex that was activated by bimodal stimuli and that showed subadditive responses to congruent (but not to incongruent) bimodal speech stimuli (Figure 2, Table 1). Unfortunately, relatively little is known about the conditions leading to this vlFC activation. Some researchers suggested that it reflects cognitive demands associated with the multisensory task such as task difficulty and memory retrieval (Beauchamp et al., 2004b). Alternatively, it might reflect rehearsal processes or motor-related activity (Meyer et al., 2011; Wuerger et al., 2011). However, our results and the study by Hein and colleagues (Hein et al., 2007) showed vlFC modulation even with task-irrelevant multimodal stimuli. Therefore, its primary role could be multisensory binding of meaningful or communication-related (speech) signals (Taylor et al., 2006). Tracer studies in monkeys observed axonal projections from the anterior STG/STS to the ventrolateral frontal cortex and from posterior STG/STS to the dorsolateral frontal cortex (Romanski et al., 1999). Our tracking results showed connections between the STC and dorsal parts of the frontal cortex. However, we observed no detectable direct tracks between posterior parts of the STG (H or PT) or other parts of our STC network with the vlFC. Instead, vlFC connections to the multisensory STC network seem to be indirect (e.g., via dlFC).

## Limitations

Although combining functional MRI and fiber tracking based on diffusion-weighted MRI provides relevant information that goes beyond the information provided by either method alone, both methods suffer from limitations that should be considered (Ramnani et al., 2004; Wakana et al., 2007; Damoiseaux and Greicius, 2009; Beer et al., 2011b). For instance, fiber tracks as determined by diffusion-weighted tensor imaging are best interpreted as white matter paths of least diffusion hindrance. Therefore, white matter tracks may result from axonal bundles (tracts) following these paths, but may also result from other sources. Moreover, tracks as determined by DWI bear no directional information as implied by the term “seed”. Seed points in tractography do not specify the origin of the fibers (e.g., cell soma) but instead an anchor for the tracking algorithm. Seed points may equally well be the targets of fibers (e.g., synapses). Similarly, connectivity cascades (e.g., from auditory cortex to msSTS via aSTS) revealed from white matter tracks must be interpreted with caution. Tracks converging at a region may result from axonal fiber bundles targeting neural populations that are separate or only polysynaptically linked (rather than monosynaptically). Furthermore, the large volume effect and crossing fiber architectures may produce inaccurate tracking results. In order to address these problems, a probabilistic (rather than deterministic) tracking algorithm was applied. This algorithm describes each voxel by its probability of being connected to the seed region. Track probabilities are a descriptive measure and the validity of tracking inferences depends on the choice of an adequate track probability threshold (Morris et al., 2008). In light of these limitations, tracking results must be reproduced across subjects (Glasser and Rilling, 2008), across studies (Wakana et al., 2007) and—if possible—across fiber tracing methods (Catani et al., 2003) in order to gain credibility. In our study tracking was performed on a group of ten brains. Consistent estimates were found across brains for most tracks. Moreover, the H and PT track estimates essentially replicated our previous findings on an independent sample (Beer et al., 2011b).

## Conclusion

In summary, functional MRI revealed a network of brain regions primarily within the temporal and occipital cortex involved in multisensory object and action processing including the msSTS region, a speech-selective lSTG region, and the EBA. Probabilistic tracking revealed white matter tracks between the auditory and the medial occipital cortex. However, brain areas involved in multisensory processing within the superior temporal and lateral occipital cortex showed little direct connectivity with primary sensory cortices. Instead these brain areas were connected to early sensory cortices via intermediate nodes of the STS and IOC. Our findings suggest that combining structural connectivity and functional imaging reveals mechanisms related to multisensory integration that remain undetected by either technique alone.

### Conflict of interest statement

The authors declare that the research was conducted in the absence of any commercial or financial relationships that could be construed as a potential conflict of interest.
